# Optimization of Starch–Tannin Adhesives for Solid Wood Gluing

**DOI:** 10.3390/polym16121694

**Published:** 2024-06-14

**Authors:** Annalisa Magnabosco, Illya Kulyk, Maurizio Avancini, Primož Šket, Jonas Eckardt, Emanuele Cesprini, Francesco Marinello, Gianluca Tondi

**Affiliations:** 1 TESAF Department, University of Padua, Viale dell’Università 16, 35020 Legnaro, PD, Italy; annalisa.magnabosco@unipd.it (A.M.); protoplasma17@gmail.com (I.K.); maurizio.avancini@studenti.unipd.it (M.A.); jonasraphael.eckardt@phd.unipd.it (J.E.); emanuele.cesprini@phd.unipd.it (E.C.); francesco.marinello@unipd.it (F.M.); 2 Slovenian NMR Center, National Institute of Chemistry, 1000 Ljubljana, Slovenia; primoz.sket@ki.si

**Keywords:** green adhesives, solid wood, surface activation, atmospheric plasma, polyphenols, shear strength, thermo-mechanical analysis, water resistance

## Abstract

Bio-based solutions for solid timber gluing have always been a very sensitive topic in wood technology. In this work, we optimize the gluing conditions of a starch–tannin formulation, which allows high performance in dry conditions and resistance to water dipping for 3 h, allowing for the D2 classification to be reached according to EN 204. It was observed that the starch–tannin formulations enhanced their performance by increasing the heating temperature, achieving satisfactory results at 140 °C for 13 min. The proportion of polyphenols in the mixture enhances the water resistance but is only tolerated until 20–30%. In particular, the addition of 10% tannin–hexamine enhances the water-resistant properties of starch for both quebracho and chestnut extract. The application of the jet of cold atmospheric plasma allows for good results with more viscous formulations, increasing their penetration in wood. Solid-state ^13^C-NMR analysis was also performed, and the spectroscopic information suggests establishing a coordination complex between starch and tannin.

## 1. Introduction

In recent years, there has been a notable shift in the landscape of adhesive technology, driven by a growing awareness of environmental sustainability and the desire to reduce the ecological footprint of various industries. Within this context, bio-based adhesives have emerged as a focal point, particularly in the domain of wood product manufacturing. These adhesives, derived from renewable resources such as agricultural and forest products, hold significant promise as a greener alternative to traditional synthetic adhesives [1,2].

The application of bio-based adhesives in gluing wood products has gained considerable attention due to their inherent eco-friendly characteristics. Comprising components extracted from nature, such as carbohydrates, proteins, lignins, and tannins, they not only offer an environmentally conscious alternative but also exhibit commendable potential for effectively bonding wood products [3]. As the demand for sustainable materials continues to rise, the exploration and utilization of bio-based derivates in the wood industry have become central to the discourse on achieving eco-friendly and resilient practices. Interesting results can be obtained in the manufactory of engineered wood products (EWPs) with small wooden fractions (fibers, particles, strands, veneer) applying bio-based adhesives. These composites often reach international regulatory standards and can be proposed as an alternative to traditional synthetic adhesives [4,5,6,7], especially for dry applications [8,9]. EWPs encompass a variety of composites created by binding different timber fractions—such as lamellas, veneer, strands, flakes, particles, and fibers—with adhesives to produce uniform elements of specific dimensions. Their widespread adoption is driven by several advantages, including the utilization of smaller wood fractions, repurposing waste wood from other manufacturing processes, minimizing defects, enhancing homogeneity, tailoring properties to specific needs, and enabling the production of diverse shapes and dimensions [10]. As a result, EWPs are becoming increasingly prevalent in a wide range of applications [11]. 

Differently, timber has a longstanding history as a structural building material, showcasing remarkable longevity when appropriately designed and maintained over centuries. In the construction sector, timber holds a well-established reputation as a pivotal material [12]. However, gluing solid timber with bio-based formulations is one of the more challenging topics in our field, highlighting the major limitations of bio-adhesives for gluing structural solid wood. Indeed, the water sensitivity of these adhesives is high, and their performance is compromised. Wood is also a highly hygroscopic material, and its dimensions also depend on water. This problem of dimensional instability further contributes to understanding the complexity of the wood–adhesive system. Thus, while the exploration of bio-adhesives for EWPs presents diverse and promising avenues [3,13,14], efforts to harness natural resources for resin production, specifically for bonding solid wood, have encountered challenges, marking a notable setback in the advancement of structural wood applications. Furthermore, it is noteworthy that the bibliography lacks extensive exploration of the utilization of bio-adhesives specifically for bonding solid wood [1,15].

Among the various bio-resources explored in the previous decades, starch-based adhesives have attracted the interest of many research groups. Their results have often led to considering this resource as an encouraging and viable solution for wood gluing [16,17]. However, these adhesives suffer intense water sensitivity, and their application potential depends on the addition of crosslinkers or on their chemical modification [18,19,20].

Starch has the advantage of being a readily available carbohydrate source [21] and has also already been studied in combination with other bioresources such as citric acid, silica, and montmorillonite to produce adhesives [22,23,24,25]. The presence of inorganics enhances the water-resistance performances of the starch adhesive, and the esterification through citric acid even guarantees an improvement after exposure to boiling water.

Following a previous work of Prof. Pizzi, which presented an interesting adhesive for particleboards [26], in the current study, we aim to develop a bio-adhesive for gluing solid wood, combining the properties of starch and tannin.

The idea of using tannin as a phenolic counterpart is intriguing because of the properties of these bio-based adhesives, which are also water-resistant when crosslinked [27,28]. 

Tannins are mainly divided into two principal classes: condensed and hydrolyzable. The structure of the former consists of flavonoid oligomers with a variable degree of polymerization, while hydrolyzable tannins are simple esters of gallic acid that are categorized according to the products obtained after hydrolysis: gallic tannins (composed of gallic acid and glucose) and ellagic tannins (composed of diaryl units and glucose) [29]. Hence, a phenolic component from both classes was examined within the formulation: quebracho extract for the condensed tannins and chestnut extract for the hydrolyzable tannins.

Cold atmospheric plasma is an ecological and economical technology that modifies the surface properties of the materials [30]. This technology allows for cleaning and activating (hydrophilic property) the treated surface, and in many cases, it also promotes the bonding of the treated surface with adhesives, coatings, and lacquers. Ignition of atmospheric plasma generates gas ions and gaseous radicals such as O, N, OH, NO, etc., which can be applied to three-dimensional objects through a plasma jet (remote plasma source) [31]. 

The idea of this research is to try to combine the starch adhesives, denatured in an alkaline environment, with tannins that can create insoluble adducts and also self-polymerize (with or without hardeners) to produce an effective bio-based adhesive for solid wood gluing. In other words, the hypothesis is to apply a more water-resistant bio-polymer, such as the tannin–hexamine, to denatured starch to create a more compact networked system through the establishment of new covalent bonds or by supramolecular encaging of the starch, which would allow an improvement of the resistance of the polymer to water.

Cold atmospheric plasma was also applied to improve the performance of the joint. 

## 2. Materials and Methods

### 2.1. Materials

For the preparation of the adhesives, the following products were used: corn starch (Maizena, Westchester, IL, USA); Tupasol ATO—quebracho (*Schinopsis balansae*) tannin extract (Silvateam, S. Michele Mondovì, Italy); Fintan C—chestnut (*Castanea sativa*) tannin (Silvateam, S. Michele Mondovì, Italy); sodium hydroxide (Alfa aesar, Haverhill, MA, USA); and hexamine (Thermofisher scientific, Waltham, MA, USA). The wooden substrates were commercial beech (*Fagus sylvatica*) lamellas of 140 × 20 × 5 mm^3^ purchased by Leroy Merlin (Vicenza, Italy) and used after conditioning. 

### 2.2. Preparation of the Adhesive

The formulation prepared in this study was obtained by adding starch to deionized water under mechanical stirring until complete dissolution. Then, the tannin extract portion was added while vigorously stirring. When needed, a water solution of 33% hexamine was added during this phase. Finally, NaOH (33% sol.) was added, and the formulation was kept under stirring for 10 min.

In Table 1, the formulations considered in this work are summarized.

### 2.3. Plasma Treatment of the Wooden Surfaces

In order to improve the performance of the adhesives, three plasma activations of the surface were performed by applying energies of 85, 255, 280, 425, and 531 J/cm^2^. The plasma was applied, exposing the wooden samples at 3 mm distance from the plasma jet, which was moved along the sample at a linear speed of 70 cm/s. For enhancement of hydrophilic property and for optimization of bonding of beech wood with starch–tannin glue, an AC cold atmospheric plasma jet MEF 1K was applied (Tigres GmbH, Marschacht, Germany), with power 250 W, generation frequency 20 ÷ 50 kHz, and compressed air (6 bar) flux 5 SLPM.

### 2.4. Sample Glueing

The wooden samples were glued by applying 170 g/m^2^ of adhesive on one lamella, and the second lamella was laid on top of the glued one, applying a slightly manual pre-pressing before undergoing the pressing stage. The samples were pressed in a Bologna presse ProtGT (Bologna, Italy) hot-press, applying a pressure of 0.1 bar for different times (3, 8, and 13 min) and temperatures (90, 110, 140, and 170 °C). After gluing, the samples were conditioned for one week before testing. 

### 2.5. Mechanical Shear Test

The samples were cut according to EN205 [32], and the shear strength was tested in the universal testing machine Galdabini Quasar 25 (Galdabini, Cardano-Varese, Italy), applying a displacement rate of 2 mm/min.

### 2.6. Water Exposure

The formulations that reached sufficiently high mechanical performance in a dry environment were prepared again, submerged in cold water for 3 h, and then allowed to dry again for 1 week before testing.

### 2.7. TMA—Thermomechanical Analysis

The adhesives with 10% quebracho and 10% chestnut (2 and 11 of Table 1), as well as formulation much richer in quebracho (50%), were tested with a Mettler–Toledo TMA/SDTA840 (Mettler Toledo, Columbus, OH, USA) to understand their behavior at different temperatures. This test was performed by applying a few mg of adhesive between two beech veneer layers of 15 × 5 × 1.5 mm^3^ and exposed to the closed chamber in which the temperature was raised from room temperature until 200 °C with a rate of 10°/min, applying a 12 s cycle of 0.1/0.5 N.

### 2.8. Chemical Investigation

The components of the formulation at different proportions were cured at 140 °C for 13 min in an oven and then left to dry in a room environment before being ground and sent for analysis. 

^13^C CP-MAS NMR spectra of solid samples were recorded on a Bruker Avance NEO 400 MHz NMR (Bruker BioSpin, Rheinstetten, Germany) spectrometer equipped with 4 mm CP-MAS probe. All samples were spun at the magic angle with 10 kHz at 25 °C. The ^1^H-^13^C CP-MAS NMR experiments consisted of excitation of protons with a 90° pulse (P1) of 3.5 μs, CP block of 2 ms, and signal acquisition with high-power proton decoupling. A total of 13,312 scans were accumulated with a repetition delay of 3 s. The chemical shifts were referenced externally using adamantine.

## 3. Results and Discussion

### 3.1. Dilution Effect

**Figure 1:**
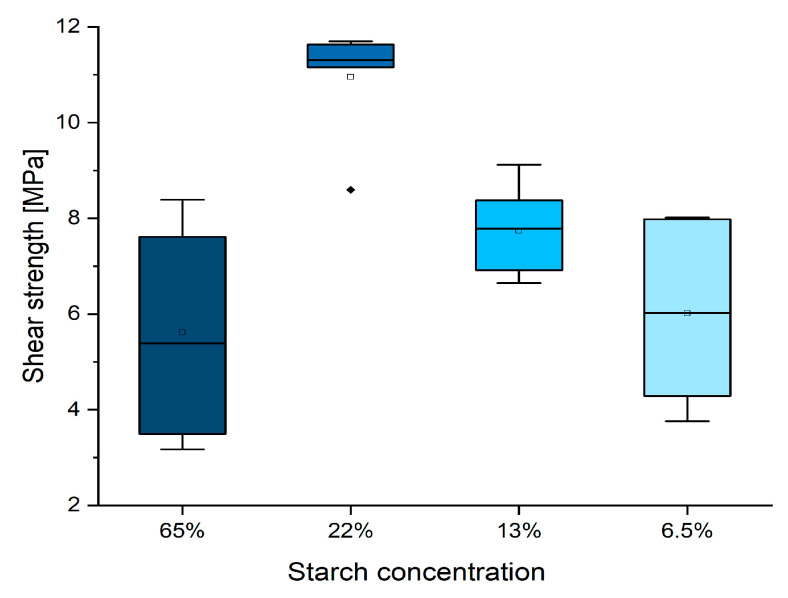
Box plot showing the dilution effect on the shear strength of the original starch–tannin formulation. (Diamonds are outliers measurements).

The preliminary study performed with the original starch–tannin formulations presented by Moubarik et al. [5] and its dilutions (formulation 1–4 of Table 1) focused on the understanding of the dilution effect on the shear strength performance of the joint after pressing the wooden specimens for 13 min at 170 °C (Figure 1).

During the distribution of the adhesive, it was observed that the viscosity strongly reduced from the original thick solution until the 3×, 5×, and 10× diluted versions. It was observed that diluting the formulation three-fold allowed for higher and more homogeneous performances. However, further dilution worsens the results due to the reduced amount of solid adhesive applied.

### 3.2. Effect of the Temperature and Time

**Figure 2:**
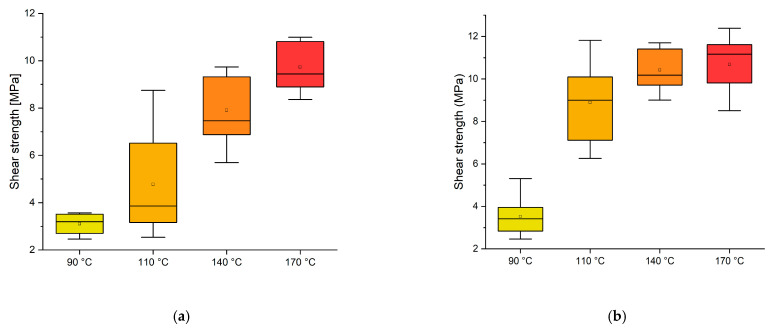
Box plot showing the effect of temperature on the shear strength: (**a**) 8 min; (**b**) 13 min curing time.

It is well-known that the curing conditions are fundamental to determining the quality of the bonding. In Figure 2, the shear performances of the adhesives were tested at different temperatures (90, 110, 140, 170 °C) and times (8 min (a) and 13 min (b)). The experiments at 3 min pressing time did not achieve a stable gluing regardless of the temperature applied.

Considering the target value of 10 MPa as the shear strength threshold for D1 adhesives according to the EN 204 [33], this experiment highlighted that at a temperature of 170 °C, the adhesive does not perform well enough with an 8 min press time. However, by extending the press time to 13 min, the normative requirement can be achieved even at 140 °C. 

### 3.3. Effect of Plasma Treatment

Despite the adhesive properties of the diluted formulations reaching the normative requirement, the amount of energy required was significant, and therefore, we tried to enhance the gluing by applying different plasma treatments on the surface before bonding.

**Figure 3:**
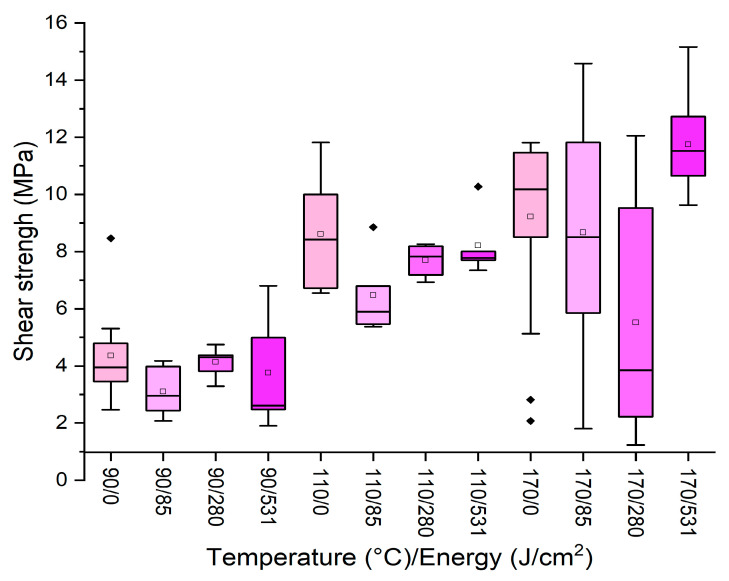
Box plots showing the effect of plasma treatment on formulation with 22% starch concentration at different temperatures. (Diamonds are outliers measurements).

In Figure 3, the effect of three different energy treatments is reported for different temperatures (90, 110, and 170 °C), maintaining a starch dilution of 22% and a pressing time of 13 min. 

**Figure 4:**
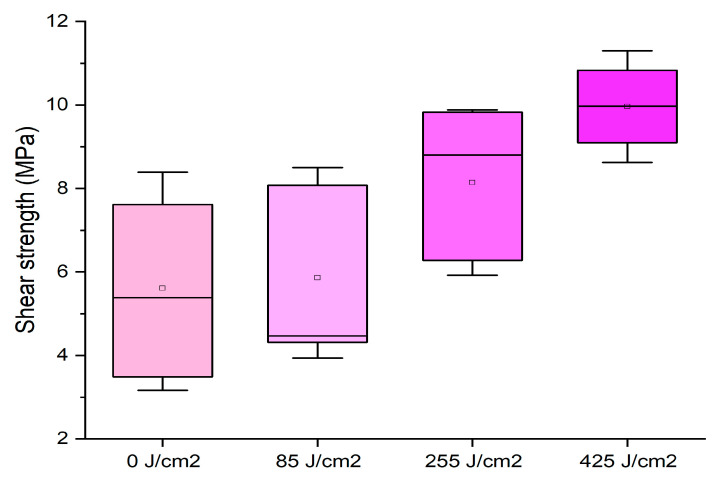
Box plots showing the effect of plasma treatment on formulation with 65% starch concentration at different surface activation energies.

It can be observed that none of the energy applied enhanced the performances registered for the untreated sample, and, in some cases, especially at high press temperature, the distribution of the data is extremely variable. Conversely, when the original adhesive was used without dilution (65% starch concentration) at 170 °C for 13 min, a significant improvement was registered by applying plasma, as reported in Figure 4.

Here, highly energetic surface treatments of 255 and 425 J/cm^2^ allowed for reaching values very close to the normative requirements. This result was due to the major penetration of the thicker adhesive, confirming that the plasma treatment is effective on the wooden surface and can be suitable to optimize the drying time of the glue joint. A similar effect was found by applying a cold plasma treatment to white lauan wood glued with phenol–formaldehyde adhesives and to beech with urea–formaldehyde resins [34,35].

### 3.4. Effect of Tannin Concentration

**Figure 5:**
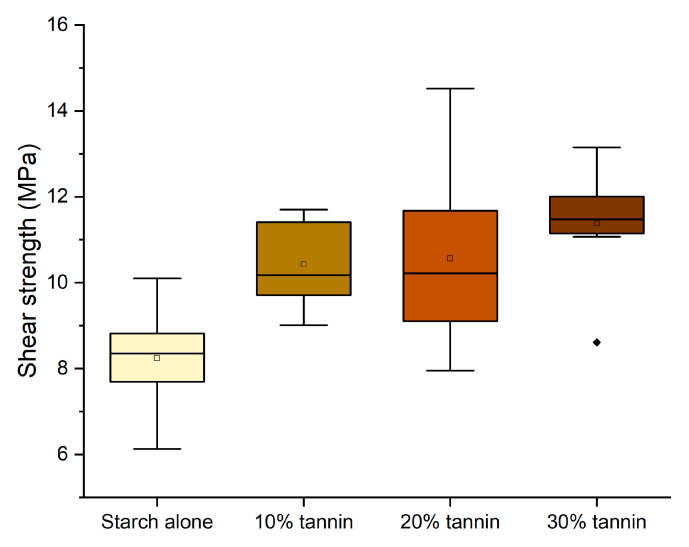
Box plots showing the effect of the tannin addition on the shear strength performance. (Diamonds are outliers measurements).

Once the more effective dilution (22%), temperature (140 °C), and curing time (13 min) were determined, we tested the effect of tannin in the starch-based formulation. In Figure 5, the shear strength of formulations containing different amounts of quebracho tannin–hexamine is tested.

**Figure 6:**
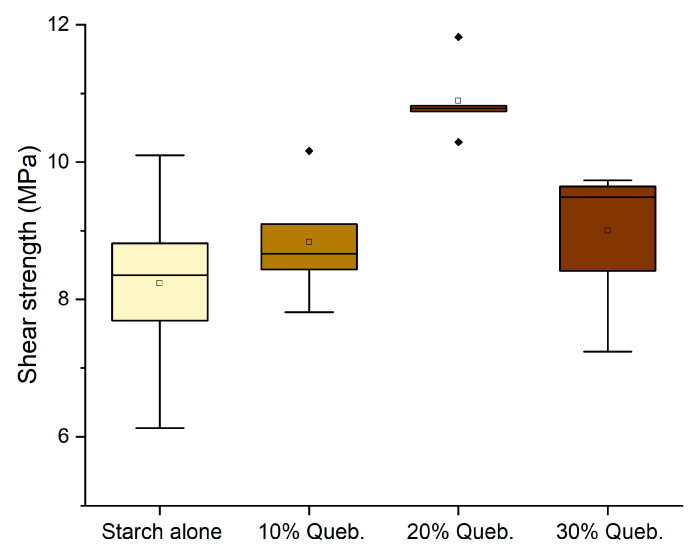
Box plots showing the Effect of the addition of hexamine-free tannin on the shear strength performance. (Diamonds are outliers measurements).

The performance remained significantly above the normative requirement for D1 adhesives (10 MPa), and therefore, we have considered pursuing the purpose further to prepare an adhesive that is perfectly bio-based (removing hexamine) and more sustainable (replacing quebracho with chestnut tannin). The tests performed without hexamine have shown that the effect of hexamine in a dry environment only slightly affects the resistance of the adhesives (Figure 6); however, this modification already worsens the performance of 10% and 30% enough to render them unsuitable as D1 adhesives. 

**Figure 7:**
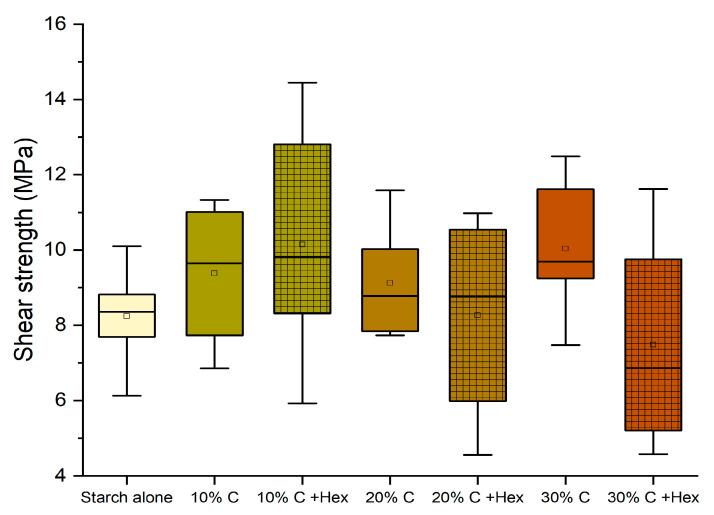
Box plots showing the effect of the addition of chestnut tannin as a polyphenolic counterpart in the shear strength performance of the adhesives.

When substituting quebracho with chestnut tannin extract, the results highlighted a similar trend to that of quebracho. However, only the 10% formulation with hexamine and the 30% without hexamine presented values slightly higher than 10 (10.14 MPa and 10.03 MPA, respectively, hence making them suitable as D1 adhesives) but with a significant data variance (Figure 7).

### 3.5. Performance after Water Exposure

**Figure 8:**
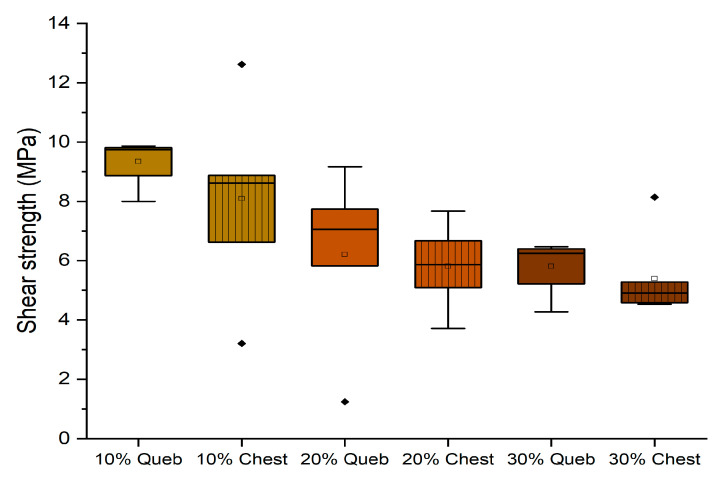
Box plots showing the shear strength performance of starch–tannin formulations without hexamine after 3 h water dipping. (Diamonds are outliers measurements).

The experiments conducted in a dry environment allowed for determining which formulation can be considered an adhesive for internal purposes (D1). It was interesting to observe the results of the adhesives after 3 h of cold water exposure to evaluate the possibility of formulating an adhesive that can tolerate water contact (D2). In Figure 8, the results of the adhesives with quebracho and chestnut without hexamine are reported. 

It can be observed that increasing the concentration of tannin involves a strong decrease in performance in a wet environment. The quebracho formulations were more resistant, probably because the condensed structure of its tannin is less hydrophilic than that of hydrolyzable ones. However, only the formulation with 10% tannin can tolerate the water, but neither quebracho nor chestnut without hexamine resulted in being suitable D1 adhesives in the dry experiments.

**Figure 9:**
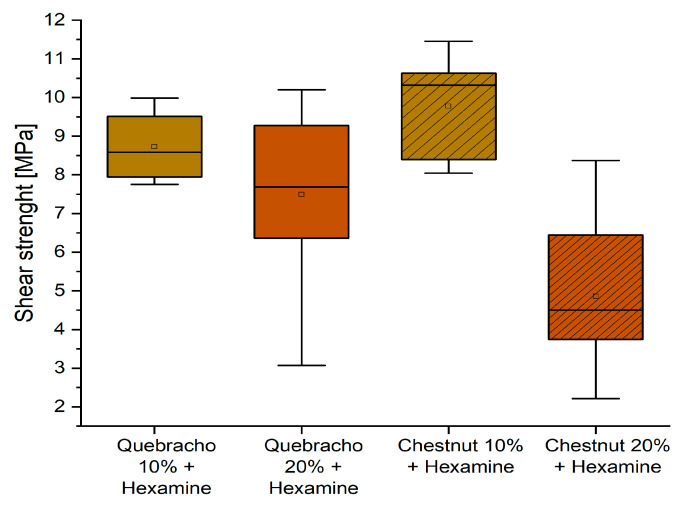
Box plots showing the shear strength performance of starch–tannin formulations with hexamine after 3 h water dipping for quebracho and chestnut tannin at 10 and 20% concentration.

Conversely, the behavior of the formulation containing hexamine is summarized in Figure 9.

It can be observed that the formulations containing 10% tannin perform better than those with 20%, and for both tannin extracts, the shear strength is higher than the required normative requirement of 8 MPa. This promotes these two formulations as being suitable as D2 adhesives, according to EN 204.

In Table 2, the most performing formulations are reported and classified according to EN 204.

These results are pretty interesting compared with the previous starch-based adhesives. Considering the modified starch, the more interesting results were obtained by preheated [36] and oxidized starch [20]. The shear strength of the starch formulation preheated at 90 °C allowed for reaching average values of 10.17 MPa in a dry environment, while the resin oxidized with hydrogen peroxide reached values of around 8 MPa when coupled with silane, and these adhesives were also resistant after water exposure to around 4 MPa. Other modifications with the objective of crosslinking the carbohydrate polymer were performed with polyisocyanate and succinic anhydride [37,38]. The former allowed for significant enhancement in dry environments (>10 MPa) and average wet shear resistance (4 MPa) after water exposure. The modification through succinic anhydride also enhanced the shear strength, but the author registered shear strength < 3 MPa. 

It may be worth noting that sodium dodecyl sulfate was also added to the adhesive, but this additive decreased the mechanical resistance of the joint; however, it allowed for better performances when aged [39].

The most interesting result was produced with inorganic fillers because silica, and especially montmorillonite, allowed for the increase in shear strength up to 5 and 10 MPa, respectively, in dry environments and 3 and 4 MPa after water exposure [24,25]. 

In this context, the results achieved with the starch–tannin formulation of this work are excellent, and this could be due to the particular arrangement of the starch in the presence of tannin that might be similar to that of the dispersion of starch and montmorillonite (5%) proposed by Li et al. [25]. Despite the outstanding result obtained, the data distribution in this work is more variable than the other studies.

### 3.6. Thermomechanical Analysis

**Figure 10:**
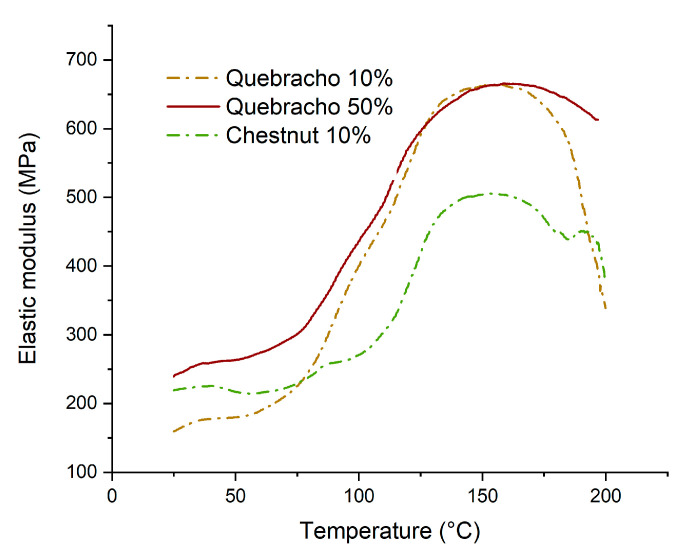
Thermomechanical analysis of starch–tannin formulations.

In Figure 10, the behavior of the modulus of elasticity of the starch–tannin adhesives is depicted. The starch-based formulations with 10% quebracho and chestnut (2 and 11, Table 1) were observed, and specifically made formulations with 50% tannin were prepared to highlight the effect of tannins.

It can be observed that the modulus of elasticity increases from 75 °C to 170 °C, and this highlights the importance of energy for the curing of these resins. In particular, at a temperature of 140–150 °C, the completeness of the curing is found, suggesting that the selected curing conditions were optimal for these resins. When quebracho is used as a source of polyphenols, the curing rate is faster than for chestnut, and the increased concentration does not affect the maximum elastic modulus. Tannin acts as a sort of catalyst for starch curing. Conversely, the formulation with chestnut produces weaker networks compared to that of quebracho (505 MPa vs. 665 MPa).

### 3.7. Chemical Investigation

^13^C-NMR were performed on the starch–tannin formulations to understand how their interaction could justify the enhancement of the mechanical properties of the joint in dry and wet environments. 

**Figure 11:**
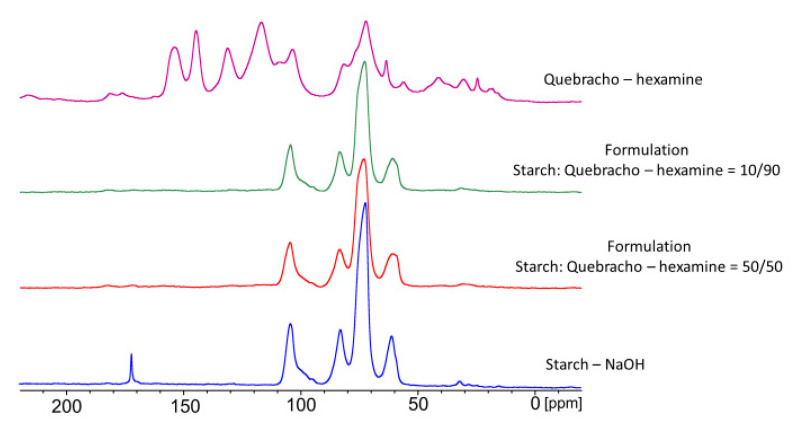
Solid state ^13^C-NMR spectra of starch and tannin–hexamine cured resin in different proportions: Pristine starch; starch:quebracho–hexamine = 50:50; starch:quebracho–hexamine = 10:90; tannin–hexamine polymer.

The ^13^C-NMR analysis was conducted on three different proportions of tannin/starch in order to highlight possible interactions between the two bio-resources during curing, and the spectra are reported in Figure 11.

It can be observed that in the mixed formulations, the signals of the starch are far more intense than the one of the tannin–hexamine. It was observed that the presence of even a contained amount of starch (10%) hides the signals of tannins (90%). This test was also repeated several times at other concentrations; therefore, we suspect that the starch shields the polyphenols, which might suggest a coordination effect of the tannin of the starch. Considering that the amount of tannin added in the formulation is never higher than 30%, this may explain the role of tannins in the formulation that act as a coordination compound for starch, modifying its structural organization of starch. 

Also, in other studies, the difficulties in registering the signals of tannin in the presence of tannin occurred despite the presence of the phenolic resin, rendering the detection of tannin more complex due to their similar chemistry [5]. Another study of the same group was more successful in observing the tannin signal; however, no details were reported on the proportion between substances [26].

## 4. Conclusions

In this work, starch–tannin formulations were optimized in order to be suitable for solid wood gluing, and the main findings are disclosed as follows:

(i) Starch–tannin adhesives from previous studies can reach the D1 classification according to EN205 when diluted at 22% by using 13 min and 140 °C or more as press conditions.
(ii) When used as a concentrated resin (65%), intensive plasma treatment (425 J/cm^2^) of the wooden substrate is required to enhance the bonding strength.
(iii) Enhancing the amount of tannin until 30% can be beneficial independently on the tannin type (quebracho or chestnut) and on the presence of hexamine as a hardener. D1 classification (10 MPa) was achieved by six formulations.
(iv) The presence of hexamine in 10% quebracho and chestnut formulations also allowed for reaching the D2 classification when testing these adhesives after leaching (3 h). These values were never registered previously for starch-based adhesives.
(v) Starch–tannin adhesives are thermosetting adhesives that start curing at around 75 °C, reaching their maximum at around 150 °C, but they start to lose their properties if exposed to excessive heat (>170 °C). Quebracho tannin enables faster and tougher curing than chestnuts.
(vi) Solid state ^13^C-NMR spectroscopy could not highlight any newer signal between starch and tannin, but a possible coordination effect is suspected due to the impossibility of significantly detecting the polyphenols when in the presence of starch. The hypothesis of having new covalent bonds could not be proven, but the coordination observed could be the main reason for the extension of the water resistance of some of the tannin-containing starch formulations. Indeed, only formulations containing hexamine were able to reach the D2 level, meaning that this crosslinking agent plays a major role in the tightness of the glue line.

These findings promote the starch–tannin adducts as a possible way for future bio-based adhesives for indoor applications, highlighting the advantages and the drawbacks of these substances. Most of the starch–tannin formulations analyzed presented a broad standard distribution of the shear strength values and high sensitivity to water; therefore, further studies are planned to improve the gluing/curing process and to further improve the water resistance. Further investigations are also planned to shed light on the chemical interactions occurring between starch–NaOH and tannin–hexamine.

## Figures and Tables

**Table 1:** Starch–tannin formulations presented in this study.

Formulation	Starch (g)	Tannin * (g)	Hexamine (g)	NaOH (g)	Water (g)
1	6.5	0.65 Q	0.1	5	10
2	6.5	0.65 Q	0.1	5	30
3	6.5	0.65 Q	0.1	5	50
4	6.5	0.65 Q	0.1	5	100
5	7.15	0	0	5	30
6	5.85	1.3 Q	0.2	5	30
7	5.2	1.95 Q	0.3	5	30
8	6.5	0.65 Q	0	5	30
9	5.85	1.3 Q	0	5	30
10	5.2	1.95 Q	0	5	30
11	6.5	0.65 C	0.1	5	30
12	5.85	1.3 C	0.2	5	30
13	5.2	1.95 C	0.3	5	30
14	6.5	0.65 C	0	5	30
15	5.85	1.3 C	0	5	30
16	5.2	1.95 C	0	5	30
17	6.5	0.65 Q	0.2	5	30
18	6.5	0.65 C	0.2	5	30
19	5.85	1.3 Q	0.4	5	30
20	5.85	1.3 C	0.4	5	30

* Q = quebracho extract; C = chestnut extract.

**Table 2:** Summary of adhesive performance and classification of the resin according to EN 204.

Adhesive Formulations	Dry Shear Strength (MPa)	Wet Shear Strength (MPa)	EN 204 Classification
2–10% Quebracho + hexamine	10.42 (0.95)	8.73 (0.99)	D2
11–10% Chestnut + hexamine	10.14 (2.65)	9.77 (1.48)	D2
7–30% Quebracho + hexamine	11.12 (1.17)	5.99 (1.89)	D1
6–20% Quebracho + hexamine	10.93 (1.95)	7.49 (2.29)	D1
9–20% Quebracho (no hexamine)	10.89 (0.56)	6.20 (3.03)	D1
16–30% Chestnut (no hexamine)	10.03 (1.78)	5.39 (1.37)	D1
14–10% Chestnut (no hexamine)	9.37 (1.82)	8.09 (3.09)	-
15–20% Chestnut (no hexamine)	9.12 (1.47)	5.81 (1.36)	-
10–30% Quebracho (no hexamine)	9.00 (0.98)	5.81 (1.03)	-
8–10% Quebracho (no hexamine)	8.83 (0.87)	9.34 (0.89)	-

## Data Availability

The original contributions presented in the study are included in the article, further inquiries can be directed to the corresponding author.
